# Supplementary material for: The Systems Biology Research Tool: evolvable open-source software
Source: BMC Syst Biol. 2008 Jun 29;2:55. doi: 10.1186/1752-0509-2-55 (PMC2446383; doi:10.1186/1752-0509-2-55)
Supplement: Additional file 1 — SBRT Archive. An archive of the current version of the Systems Biology Research Tool. [file 1752-0509-2-55-S1.zip › sbrt-1.4.0/doc/developers_guide/api/sbrt/kernel/fba/comp/class-use/FluxomeReducer.html]

Uses of Interface sbrt.kernel.fba.comp.FluxomeReducer


|  |  |  |
| --- | --- | --- |
| **Overview** \| **Package** \| **Class** \| **Use** \| **Tree** \| **Deprecated** \| **Index** \| **Help** | |  |
| PREV   NEXT | **FRAMES**    **NO FRAMES**     **All Classes** |  |


---


## **Uses of Interface sbrt.kernel.fba.comp.FluxomeReducer**

| Packages that use FluxomeReducer | |
| --- | --- |
| **sbrt.kernel.fba.comp** | Provides classes and interfaces for performing Flux Balance Analysis computations. |
| **sbrt.kernel.fba.proc** | Provides classes and interfaces for executing Flux Balance Analysis processes. |

Uses of FluxomeReducer in sbrt.kernel.fba.comp

| Classes in sbrt.kernel.fba.comp that implement FluxomeReducer | |
| --- | --- |
| `class` | `MsFluxomeReducer`             This class is used to reduce the size of fluxomes for the purpose of identifying the steady state cycles (Type III extreme pathways) they contain. |
| `class` | `WwFluxomeReducer`             This class is used to reduce the size of fluxomes for the purpose of identifying the steady state cycles (Type III extreme pathways) they contain. |

Uses of FluxomeReducer in sbrt.kernel.fba.proc

Constructors in sbrt.kernel.fba.proc with parameters of type FluxomeReducer

`FluxomeReducerProcess(T fluxome,
FluxomeReducer<T> reducer)`
  
          Constructs a new fluxome reduction process.

---


|  |  |  |
| --- | --- | --- |
| **Overview** \| **Package** \| **Class** \| **Use** \| **Tree** \| **Deprecated** \| **Index** \| **Help** | |  |
| PREV   NEXT | **FRAMES**    **NO FRAMES**     **All Classes** |  |


---
